# Recent Developments in Graphene-Based Adsorbents for Environmental Applications

**DOI:** 10.3390/nano16140884

**Published:** 2026-07-17

**Authors:** Stelian Pintea, Adina Stegarescu, Ildiko Lung, Anda Maria Chiș, Emanuela Dana Lushnykov, Maria-Loredana Soran, Ocsana Opriș

**Affiliations:** National Institute for Research and Development of Isotopic and Molecular Technologies, 67-103 Donat Street, 400293 Cluj-Napoca, Romania; stelian.pintea@itim-cj.ro (S.P.); adina.stegarescu@itim-cj.ro (A.S.); ildiko.lung@itim-cj.ro (I.L.); anda.chis@itim-cj.ro (A.M.C.); emanuela.lushnykov@itim-cj.ro (E.D.L.); loredana.soran@itim-cj.ro (M.-L.S.)

**Keywords:** graphene-based adsorbents, surface functionalization, water decontamination, air purification, soil remediation, environmental remediation

## Abstract

Graphene and its derivatives have attracted sustained research interest as adsorbent materials for environmental applications, driven by their large surface area, chemically tunable surface, and compatibility with a wide range of functional modifications. This review covers recent developments in the use of graphene-based materials for water, air, and soil remediation, focusing primarily on work published over the last five years. A concise overview of graphene, its derivatives, and other carbon nanostructures, such as carbon nanotubes and fullerenes, is also provided. The main graphene derivatives are briefly described (graphene oxide, reduced graphene oxide, graphene nanoribbons, and graphene quantum dots) together with a comparative overview of the principal synthesis methods, from mechanical exfoliation and chemical vapor deposition to liquid-phase exfoliation, oxidation/reduction, and flash Joule heating. The discussion then turns to how surface functionalization and composite formation affect adsorption performance in practice. In water treatment, the results are most developed: functionalized composites have reached adsorption capacities of 484.3 mg g^−1^ for organic dyes and 157.23 mg g^−1^ for Cr(VI). Air purification is a smaller but growing area, with plasma-treated graphene aerogels achieving CO_2_ capture capacities of 3.3 mmol g^−1^ and retaining performance over 40 cycles. Soil remediation remains the least explored compartment, though arsenic immobilization efficiencies of up to 99.3% have been reported. Remaining challenges around scalability, behavior in real environmental matrices, and long-term ecotoxicological impact are identified and discussed.

## 1. Introduction

Two-dimensional (2D) or single-layer materials have always been considered promising materials due to their peculiar (expected/predicted) properties. More than two decades after it was first isolated in 2004 at the University of Manchester by the group of Andre Geim and Konstantin Novoselov, the name graphene is “now hundreds, maybe even thousands of different things”, to cite Mark Peplow [1].

**Figure 1:**
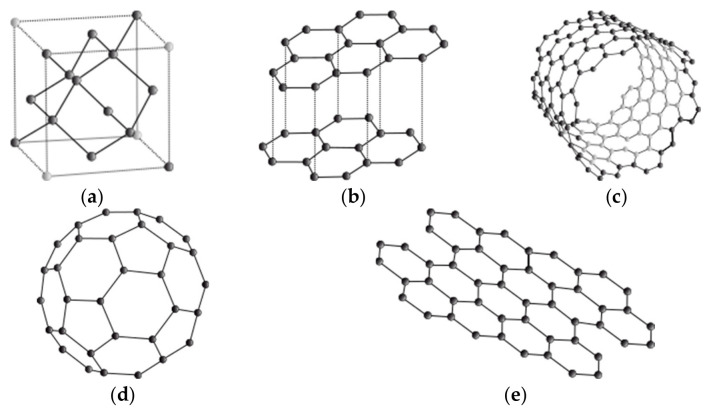
Representative carbon structures and nanostructures with different dimensionalities: (**a**) diamond, (**b**) graphite, (**c**) carbon nanotube, (**d**) fullerene, and (**e**) graphene.

Graphene belongs to a broader family of carbon nanostructures and carbon-based materials that span different dimensionalities, including zero-dimensional (0D) fullerenes, one-dimensional (1D) carbon nanotubes, two-dimensional (2D) graphene, and three-dimensional (3D) graphite and diamond, as illustrated in Figure 1 [2]. Their specific applications strongly depend on their physical and chemical properties, which are closely related to their structure at the atomic level.

Since its discovery and separation, graphene has expanded in the number and types of applications. Today, graphene and its derivatives are found in a large variety of technological and scientific applications. Examples include advanced materials, energy storage, medical devices, and electronics. Among medical applications, we would like to emphasize its use in drug delivery systems where its surface modifications are exploited. Biosensors and portable diagnosis devices could also be enhanced by the use of graphene and its derivatives. The energy storage industry is continuously developing new solutions for higher-performance batteries, making use of graphene’s high surface area that allows for quick charging and discharging processes, greater storage capacity, etc. Also, the field of electronics is flourishing with the use of graphene for better sensors, faster transistors, and more flexible displays.

In time, the number of environmental applications of graphene grew exponentially, catalyzed by the massive investments in the study of both the pristine material and its derivatives [3].

If taking into account only the last decade, the number of graphene-related scientific publications concerned with water decontamination processes has two main characteristics: fast growth in number and constant broadening of the type of materials applied in the research (as a pollutant or functionalized material) [4,5,6,7].

Air, as one of the key elements that sustains life on Earth, is also affected by pollution like in soil, water, and other parts of our environment. Moreover, air pollution seems to have a much higher propagation/diffusion speed than that of water or soil, to name the basic elements required for a clean environment. The increased production rate of the industrial era we are living in also comes with the price of rising amounts of CO_2_, NO, NO_2_, H_2_S, and SO_2_ dispersed in the atmosphere. Air pollution is known for its severe effects on human health, causing serious diseases like tumors, allergic reactions, dry eyes, cardiovascular issues, lung cancer, etc. Even CO_2_, which could be considered innocent at normal concentrations, can cause respiratory problems when its concentration increases above 7.2%. Much more severe effects are coming from the CO pollutant of air that competes with O_2_ in binding with the hemoglobin present in the blood composition, blocking the necessary O_2_ from reaching the cells forming different tissues and organs.

The interest and, subsequently, the number of applications involving the use of graphene or its derivatives increased rapidly after it was obtained for the first time in 2004. Accordingly, the number of scientific papers and reviews has also increased, each with different approaches and limited coverage, highlighting the large diversity of the applications of graphene and graphene-based materials.

This review briefly covers those applications where graphene or graphene derivatives are used to produce adsorbents with an environmental impact. The main properties and the preparation methods involved in these experiments were also briefly presented, and, where needed, the reader was redirected towards more detailed studies.

At the same time, where possible, we focused on applications from the last few years, as close as possible in time to these days. There is no shortage of reviews on graphene-based adsorbents, but most of them concentrate on one environmental compartment, usually water, or on a specific class of pollutants like heavy metals or organic dyes. What is harder to find is a single overview that treats water, air, and soil remediation together; covers the main synthesis routes with their practical trade-offs; and connects materials design to real-world performance in a consistent way. That is what this review attempts to do, drawing primarily on work published over the last five years. The synthesis methods are discussed not just in terms of what materials they produce, but also in terms of scalability and cost since those factors matter as much as adsorption capacity when thinking about actual deployment. Computational approaches, particularly density functional theory (DFT), are also covered, as they are increasingly being used to interpret the experimental results and guide the design of new composites rather than simply describe them after the fact.

The literature on graphene-based adsorbents has by now grown too large to cover in full, and attempting an exhaustive survey would not have made this review any more useful. We chose instead to concentrate on a smaller set of studies that we considered representative, and we selected them for two reasons. The first was how clearly each one shows the adsorption mechanism at work, whether through π–π stacking, electrostatic attraction, or hydrogen bonding. The second was whether the study marked a real improvement in performance for water, air, or soil, rather than adding one more result under the same conditions. Where possible, we focused on work from the last five years, partly because the strongest adsorption results discussed here come from this recent work, and partly because it is only recently that DFT has begun to be used to predict which modifications will actually help, instead of explaining results after the fact. Selecting the literature this way was meant to keep the mechanism and the underlying design principles in focus, and to bring out the directions most likely to drive the next applications, rather than to assemble an exhaustive bibliography.

## 2. Brief Historical Evolution

It was only as late as 1859 that Benjamin Collins Brodie became aware of the lamellar structure of thermally reduced graphite oxide [8,9]. The X-ray Diffraction (XRD) method was applied by Debije and Scherrer in 1916 in order to determine the atomic structure of graphite [10].

Three decades later, in 1947, a paper by Canadian physicist P.R. Wallace developed the band structure of graphite using the tight-binding approximation [11]. In 1962, Boehm reported the production of few-layer graphite, another step towards the single-layer graphite or, as we know it today, graphene [12].

The scientific attention towards graphene only appeared in the second half of the 20th century, when the higher basal plane conductivity of graphite intercalation compounds with respect to that of the original graphite was discovered [13,14]. Further on, the graphene research grew slowly for the remaining decades of the century. Both experimental observations and theoretical calculations considered graphene as a thermodynamically unstable material unless its size increased above 6000 atoms, where it would become very stable for sizes of at least 24,000 atoms [15]. Theoretical calculations predicted that graphene would only be stable if it reached thicknesses of 20 nm.

Several methods were used for trying to produce graphene; although with limited success, they still delivered materials with significant enhancement of charge mobility [16,17,18,19].

The final step towards graphene’s world glory was its isolation at the University of Manchester by Andre Geim and Konstantin Novoselov in 2004. The applications quickly showed up, leading to the production of graphene ink in 2008, transparent electrodes in 2009, and the enhancement of the technology of thin-film transistors and electrochemical sensors. The use of graphene as a transparent electrode allowed for the production of large-scale displays and solar cells based on it.

The highest international recognition for graphene came in 2010 when Andre Geim and Konstantin Novoselov won the Nobel Prize in Physics. Its trip in the scientific and technological realms followed a path that included the first graphene-based photodetectors in 2012, which revealed the ability to convert photons into an electric current. In 2014, it was found that batteries could be significantly improved by the use of graphene, too. In 2015, the first magnetic field sensor based on graphene was witnessed. In 2016, the broad range of applications improved by the use of graphene were confirmed, like mobile phones (batteries, screens), aircraft, cars, data transmission, etc. In 2017, graphene was used in the renewable energy sector: interface engineering and solar cell production. The story goes on, getting more complex, leading to a myriad of applications in our daily lives.

As one can see, after its discovery, graphene determined a rapid acceleration in the field of science and technology in order to exploit the extraordinary properties of these thin carbon layers, such as electrical, thermal, and mechanical [20]. Its use in environmental applications is not an exception to this rule. Scientists are more and more efficient in incorporating it into different composite materials and in exploiting its exceptional properties, whether referring to water, soil, or air decontamination processes.

## 3. Graphene-Based Materials/Adsorbents

The exceptional properties of graphene stimulated scientists to tailor it towards specific applications, leading to a large variety of graphene-based materials.

### 3.1. Graphene (Pristine)

Pristine graphene is the basic type of graphene adsorbent used these days. It contains a single layer of carbon atoms arranged in a hexagonal atomic structure with higher strength than steel and very good thermal and electrical conductivity [21,22]. Although obtaining it in large thin films is still challenging and expensive, the pristine form of graphene comes with valuable applications in high-tech electronic devices and various composite materials. A crucial point in its production process is to obtain a high-purity final product that maintains its fantastic properties.

### 3.2. Graphene Oxide (GO)

Graphene oxide (GO) has emerged as a graphene derivative with distinctive properties that make it suitable for various technological applications such as energy storage and conversion, healthcare, sensing, and environmental remediation [23]. It consists of a sheet of a single carbon atom layer decorated with oxygen-containing functional groups like hydroxyl and epoxy on the basal plane and carbonyl, carboxyl, and phenol groups on the edges of the carbon sheet. These groups alter their properties, like electrical conductivity, with respect to those of pristine graphene, making it more hydrophilic [24]. This is particularly helpful for liquid processes where dispersion in water is crucial. Although some of its properties are below those of pristine graphene, GO is successfully used as a precursor for other graphene-based materials and also in water decontamination processes [25].

### 3.3. Reduced Graphene Oxide (rGO)

Reduced graphene oxide, rGO, is a reduced form of graphene oxide with a closer structure to that of pristine graphene, obtained by removing the oxygen-containing functional groups. Depending on the production method, it might contain a small amount of oxygen and hydrogen. It is produced by partially reducing GO, increasing the electrical conductivity while still retaining some oxygen groups [26]. The reduction process induces defects that affect its performance, distinguishing it from both pristine graphene and GO. Nevertheless, its conductivity and hydrophilicity make it suitable for battery and supercapacitor technology [27].

### 3.4. Graphene Nanoribbons (GNRs)

As their name suggests, graphene nanoribbons are ribbons of graphene with a width of up to 100 nm. As a theoretical model, they were introduced by Mitsutaka Fujita for edge effect investigations in the case of graphene [28,29]. GNRs can be produced by graphite nanotomy, axially cutting carbon nanotubes, plasma etching nanotubes etc. [30,31]. An increase of the mechanical properties of epoxy composites as well as of the polypropylene fumarate was observed by loading GNRs or oxidized GNRs [32,33]. GNRs were reported as a contrast agent for photoacoustic and thermosacoustic imaging and tomography, as well as a good candidate for catalysts or catalyst support.

### 3.5. Graphene Quantum Dots (GQDs)

There are several types of quantum dots (QDs): inorganic QDs, organic QDs, carbon QDs, perovskite QDs, etc. Graphene quantum dots (GQDs) combine the excellent properties of graphene with those of the quantum dots [34]. Due to their biocompatibility, photostability, and tunable luminescence, they are useful in bio-imaging applications. We can also find them in cancer treatment applications where their photothermal and photodynamic properties are exploited to cause cancer cell death.

## 4. Graphene Preparation Methods

Scientists believed that 2D carbon-based materials could not exist independently because they would be thermodynamically unstable and would decompose/transform into more stable carbon structures [35]. Nevertheless, today, we have several ways to produce graphene and/or its derivatives. We will briefly discuss in the following paragraphs micromechanical cleavage, liquid-phase exfoliation, oxidation exfoliation, chemical vapor deposition, and silicon carbide-based and flash-joule heating methods for G/GO synthesis. It is important to mention that the synthesis method used to produce graphene or its derivatives will determine the properties of the product obtained. In this respect, depending on the applications in which we intend to use the prepared materials, the most appropriate method is also chosen.

### 4.1. Micromechanical Cleavage

**Figure 2:**
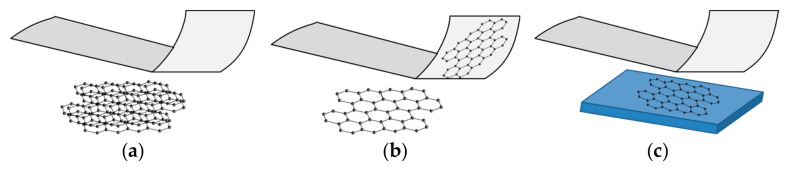
The main steps in producing graphene using the scotch tape method: (**a**) adhesion of the tape to highly ordered pyrolytic graphite, (**b**) repeated exfoliation resulting in thinner graphene layers on the tape, and (**c**) transfer of the graphene layer onto an oxidized silicon substrate and removal of the tape using solvents.

Micromechanical cleavage is the method used for the first successful attempt to produce graphene. The first experimentally produced graphene was obtained using the top-down exfoliation synthesis technique, making use of scotch tape [36]. The preparation itself consisted of the extraction of single-atom-thick crystallites from bulk graphite. The graphene layers removed from the graphite were transferred onto a silicon wafer in a process called micromechanical cleavage or, more popularly, the scotch tape method. The method only requires a graphite source and adhesive tape that continuously peels off the graphite, leading to the cleavage of thinner and thinner flakes with an almost atomically flat surface. The adhesive tape is then dissolved into a solvent (acetone), and an oxidized Si wafer is introduced into the solution in order to have some graphene flakes deposited on the wafer [3]. A short and simplified graphical presentation of the main steps of this method is depicted in Figure 2. Repeated peeling off of the adhesive tape pressed towards highly ordered pyrolytic graphite leads to the adhesion of a thinner graphene layer on the tape (Figure 2a,b). The graphene layer is then transferred to an oxidized silicon substrate (Figure 2c), where the tape is dissolved using solvents.

The previously described scotch tape method, or mechanical exfoliation, was extremely useful in proving the existence of graphene layers, but beyond the high-quality graphene it produces, it is rather unfeasible for large-scale production due to its low yield. To increase the quantity and reproducibility of the graphene production methods, scientists developed other methods, each of them having their own advantages and challenges.

### 4.2. Chemical Vapor Deposition

**Figure 3:**
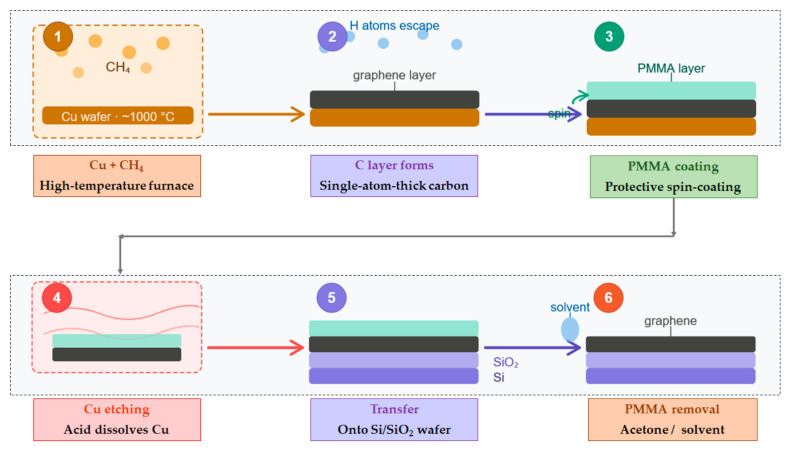
Schematic representation of the chemical vapor deposition (CVD) process for graphene production on a copper substrate: (**1**) copper (Cu) wafer exposed to methane (CH_4_) atmosphere at high temperature (~1000 °C); (**2**) carbon (C) atoms deposit on the Cu surface, forming a single-atom-thick graphene layer while hydrogen (H) atoms escape; (**3**) the graphene layer is covered with a poly(methyl methacrylate, PMMA) protective coating applied by spin-coating; (**4**) the Cu substrate is dissolved in acid (Cu etching), releasing the PMMA/graphene bilayer; (**5**) the bilayer is transferred onto a Si/SiO_2_ wafer; and (**6**) the PMMA layer is removed using acetone or another appropriate solvent, leaving the graphene film on the substrate.

Chemical vapor deposition (CVD) is a method used to produce 2D materials in general. In the case of graphene, it starts with a Cu wafer exposed to a CH_4_ atmosphere and heated to high temperatures. When touching the hot copper wafer, the C atoms of methane are trapped while the hydrogen atoms keep moving around. A tightly bonded, single-atom-thick carbon layer is produced this way. The graphene layer obtained is covered by an organic polymer (polymethyl methacrylate, PMMA) protective layer using the spin-coating method. The copper substrate is dissolved in an acid that does not react with the graphene. After washing away the remaining acid residues, the PMMA/graphene double layer is recovered using another Si/SiO_2_ wafer. Before use, the PMMA layer is removed with a specific solvent such as acetone, leaving the graphene film on the substrate. The six main steps of this process are schematically illustrated in Figure 3.

This method produces large areas and high-quality graphene but comes with the need to use specialized equipment and a relatively complex production process. Overall, it increases the yield by an order of magnitude with respect to mechanical exfoliation. The process requires good control of several parameters (temperature, pressure, gas flow) but produces good-quality and large area graphene.

### 4.3. Thermal Decomposition of Silicon Carbide

Silicon carbide (SiC) is an abrasive compound mass-produced since 1893, when it was seen as a replacement for diamond. It is also used as a wide-bandgap semiconductor. In nature, it occurs as an extremely rare moissanite mineral. The thermal decomposition of SiC leads to large surface-area graphene. The SiC heating process is performed in a controlled environment, ultrahigh vacuum, or argon atmosphere [37,38]. The surface silicon atoms’ sublimation induces a carbon-rich surface that leads to graphene layers. Strict control on heating parameters like temperature and heating duration is required for large area layers [39]. The process produces high-quality and expensive graphene layers.

### 4.4. Liquid Phase Exfoliation

Liquid-phase exfoliation (LPE) is a method used for producing graphene from bulk graphite [40]. The dispersion of graphite in a liquid medium is achieved under solvents and ultrasonication conditions [41]. Typically, the process involves the use of a N-Methyl-2-pyrrolidone (NMP) solvent or different surfactants in order to prevent re-aggregation of the graphene sheets. The exfoliated graphene is then separated by size and layer count from the unexfoliated graphite sheets via centrifugation. The resulting stable graphene dispersion can be further processed by vacuum filtration to obtain graphene films suitable for various applications [35,42].

**Figure 4:**
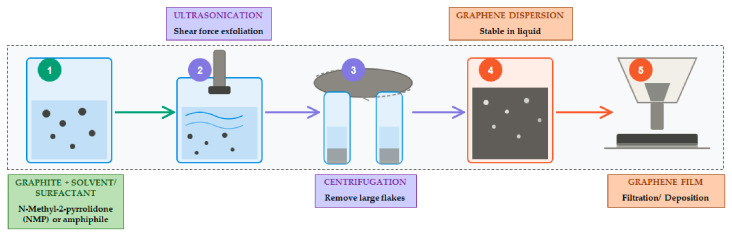
Schematic representation of the liquid-phase exfoliation (LPE) process for graphene production from bulk graphite: (**1**) dispersion of graphite in solvent/surfactant (N-Methyl-2-pyrrolidone, NMP, or amphiphile); (**2**) ultrasonication applying shear force for layer exfoliation; (**3**) centrifugation to separate exfoliated graphene sheets from unexfoliated graphite flakes; (**4**) collection of stable graphene dispersion; (**5**) filtration/deposition to obtain a graphene film.

The main steps of the LPE process are schematically illustrated in Figure 4, showing the sequential stages from graphite dispersion in solvent/surfactant (step 1), ultrasonication (step 2), centrifugation (step 3), graphene dispersion collection (step 4), and final film formation by filtration/deposition (step 5).

Although it is easily scalable, the LPE graphene production method introduces defects and contamination from the used surfactants, as well as variations in the thickness of the produced graphene. It is estimated that more than half of the global graphene production is based on LPE [43].

### 4.5. Oxidation–Reduction

The oxidation–reduction method starts with graphite oxidation and is followed by exfoliation and reduction that produces reduced graphene oxide [44]. The first step is the oxidation and exfoliation of graphite to obtain graphene oxide (GO). The GO can be reduced to produce reduced graphene oxide (rGO). The oxidation of graphite is performed using strong oxidizing agents like nitric acid, sulfuric acid, and potassium permanganate. This increases the interlayer spacing by introducing oxygen-containing functional layers such as hydroxyl, epoxy, or carboxyl [45,46]. The increased interlayer distance allows for the exfoliation of GO sheets through sonication in solvents like water. The GO produced this way is hydrophilic and lacks the conductive properties of graphene. In order to improve its electrical conductivity properties, the GO is reduced in order to restore the sp^2^ carbon structure disrupted during the oxidation step. As reducing agents, we normally use hydrazine, sodium borohydride, or thermal methods. This method allows for the production of large amounts of graphene at a low cost, but rGO often presents structural defects and oxygen group residues that lower its electronic properties with respect to pristine graphene. The quality can be improved by optimizing the reduction conditions, using environmentally friendly reducing agents, or using electrochemical reduction methods. The oxidation–reduction synthesis technique is widely popular due to its scalability and ease of process; however, minimizing the defects for specific applications is still challenging.

### 4.6. Flash Joule Heating

Another method for graphene production explored by scientists is called flash Joule heating. It involves the rapid heating and cooling of a rich-carbon-containing material (coal, food waste, etc.) in order to transform it into graphene. During the process, the carbon source is rapidly heated to ultrahigh temperatures that can rise above 3000 °C, using high-energy electric pulses that drive the rearranging of carbon atoms and their graphitization [47]. The method can optimize the structural parameters of carbon materials (interlayer spacing, defect concentration, heteroatom doping) by precise control of operating parameters (flash voltage and time). Using this method, Hosseinzadeh et al. successfully prepared graphene with customized magnetic properties, as shown in their paper from 2025 [42]. Another advantage of this method is the possibility of using any kind of carbon-rich material, making the precursors easily available.

Table 1 briefly emphasizes the main characteristics of the discussed preparation methods for graphene.

The preparation of graphene for industrial usage is still challenging, although we are more than two decades away from the moment it was first obtained by the use of a trivial scotch tape method.

Beyond these preparation methods used to produce different qualities and quantities of graphene, there are several methods that are used in order to functionalize the surface structure of graphene or to customize its properties. These methods strongly depend on the properties addressed during the functionalization and the final characteristics we want to obtain for our samples. Upscaling comes, in general, as a later problem. The priority in general is the design and precise control of the composite we want to produce. Given the large variety of functionalization methods described in the literature and the representative rather than exhaustive scope of this review, we invite the readers to look for specific papers that discuss their special interest, while further discussing some of the recent environmental applications of graphene-based adsorbent materials. Also, a more detailed cost and scalability comparison between FJH and CVD is provided in Section 9.

## 5. Graphene-Based Materials—Properties and Characterization

Like any nanometric-sized material, graphene also possesses a large specific surface area. This makes it suitable for being used, on its own or in composite materials, as an adsorbent material for environmental applications.

One of the properties exploited when designing environmental applications containing graphene or graphene-based compounds is its relatively high chemical stability, which limits its interaction with other substances. Nevertheless, as one of the most investigated 2D materials, graphene is also open for functionalization, giving a large variety of possibilities to enhance its properties [48]. The number of layers regulates the properties of graphene. In Table 2, selected characteristics are listed as a function of the number of graphene layers. Although only a few characteristics are listed, it proves that single-layer graphene (SLG) outcompetes all its competitors from the graphene family in most of the scientifically relevant properties. Practically, all the exceptional properties of the single-layered material decrease with the increase of its thickness, altering its purity.

The differences between SLG, DLG, and MLG are mainly related to the number of layers, which influences the interaction between them and, implicitly, their properties [62]. In general, SLG exhibits the highest specific surface area, which is advantageous for applications such as adsorption [63]. As the number of layers increases, partial overlapping between sheets may occur, leading to a decrease in the accessible surface area [64]. However, DLG and MLG can provide improved stability compared to single-layer graphene, which can be beneficial in practical applications [65]. In the case of graphene oxide, although some fundamental properties are reduced, the presence of functional groups may enhance its interaction with different substances [63].

Preserving these properties is costly, and scientists are trying to avoid their drop by functionalization that attenuates the decrease for specific characteristics, depending on the goal of the new material.

The drop in specific surface area, for example, could alter the adsorbing and catalytic properties of the graphene-based materials, making their use less efficient in the decontamination processes. Similar stories could be drawn for the electron mobility, thermal conductivity, or elastic properties of graphene and its derivatives.

## 6. Water Decontamination with Graphene-Based Composites

The review by Sophia et al. is a good starting point for an overview of the adsorption mechanisms involved in graphene-based materials used for water decontamination for pharmaceuticals [65]. Another review we would recommend to our readers was published by Saravanan et al. and discusses mainly the different types of graphene-based composites (graphene oxide, graphene quantum dots, graphene nanoplatelets, etc.), referring to their preparation process as well as their use as adsorbents, electrode materials, or photocatalysts [66]. By far, the most used process for water decontamination is adsorption, due to its simplicity of application, cost-effectiveness, and ease of preparing the adsorbent materials.

**Figure 5:**
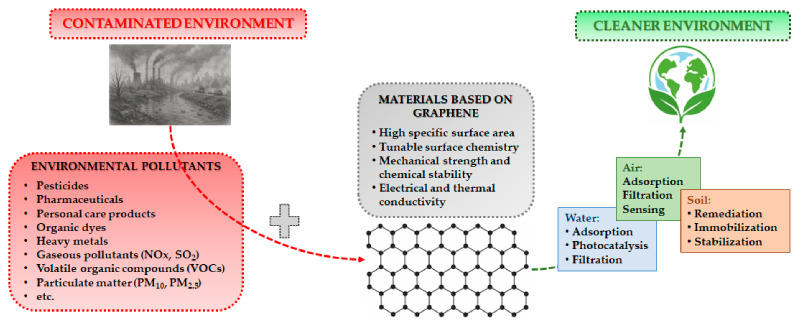
Schematic representation of graphene-based materials applied in environmental decontamination processes across water, soil, and air matrices. The left panel illustrates the main categories of environmental pollutants addressed, including pesticides, pharmaceuticals, personal care products, organic dyes, heavy metals, gaseous pollutants (NO_x_, SO_2_), volatile organic compounds (VOCs), and particulate matter (PM_10_, PM_2.5_). The central panel highlights the key physicochemical properties that make graphene particularly suited for remediation applications, namely its high specific surface area, tunable surface chemistry, mechanical strength and chemical stability, and electrical and thermal conductivity. The right panel summarizes the principal remediation strategies enabled by graphene-based materials depending on the target environmental compartment: adsorption, photocatalysis, and filtration for water treatment; adsorption, filtration, and sensing for air purification; and remediation, immobilization, and stabilization for soil decontamination.

Figure 5 is a schematic representation of graphene-based materials applied in environmental decontamination processes across water, soil, and air matrices. The left panel illustrates the main categories of environmental pollutants addressed, including pesticides, pharmaceuticals, personal care products, organic dyes, heavy metals, gaseous pollutants (NO_x_, SO_2_, etc.), volatile organic compounds (VOCs), and particulate matter (PM_10_, PM_2.5_) [67,68,69]. The central panel highlights the key physicochemical properties that make graphene particularly suited for remediation applications. Graphene-based materials have high specific surface area, tunable surface chemistry, mechanical strength and chemical stability, and electrical and thermal conductivity [48,53,59,63]. The right panel summarizes the principal remediation strategies enabled by graphene-based materials depending on the target environmental compartment: adsorption, photocatalysis, and filtration for water treatment [4,6]; adsorption, filtration, and sensing for air purification [70,71,72]; and remediation, immobilization, and stabilization for soil decontamination [73,74,75]. Together, these features position graphene-based materials as versatile and promising candidates for transitioning from contaminated to cleaner environments [76,77].

Heavy metals are often removed from aqueous environments using adsorbent materials. Their main sources are industrial human activities like mining and steel processing coating [78]. The most investigated heavy metals are As, Hg, Co, Ni, Cd, Pb, etc. As and Hg removal from aqueous environments was investigated and reported in publications by Chevinli et al. [79] and, respectively, Barra et al. [80]. In the first case, composite adsorbent materials of Mg–Fe layered double hydroxide (LDH) with GO for the removal of As(III) and As(V) were prepared and characterized. The maximum adsorption capacity of 307 mg g^−1^ was achieved for As(V) removal using LDH 0.42—80-GO20, which contains an Fe/(Fe + Mg) molar ratio of 0.42 and 80 wt% mass content of LDH. On the other hand, Barra et al. used GO foams modified with caffeic acid in order to remove Hg^2+^ from contaminated water environments. They applied the hydrothermal reduction of graphene oxide in the presence of caffeic acid (CA), which was further modified with chitosan (CS). The Hg(II) maximum sorption capacity of 2.79 mg g^−1^ was obtained for a pH value of 4–6. Furthermore, the low desorption rate and its applicability in real complex matrices are advantages of the rGO-CA that can be exploited for efficiently removing and immobilizing Hg from various aqueous environments. Regarding the specific surface area, a crucial parameter when evaluating the adsorption capacity, it was observed that the addition of CA and CS decreased the specific surface area of the pristine rGO. For other toxic heavy metal removal from aqueous solutions, making use of graphene-based materials, we refer to the very recent works of Yokwana et al. and Ozalp et al. [81,82].

Another recent investigation comes from Singh et al., published in 2024, and discusses the functionalization of a metal–oxide framework with graphene [83]. The UiO-66 framework used is a porous material that presents a uniform and large pore size; high surface area; very good water, chemical, and hydrothermal stability; and active clusters of ZrO [84,85]. The UiO-66-NDC (1,4-naphthalenedicarboxylic acid) metal–organic framework (MOF) presents Zr_6_O_4_(OH)_4_ blocks to create a three-dimensional structure with an octahedral center hole cage and eight tetrahedral corner cages [86]. The functionalization with graphene is meant to improve the low adsorption capacity and selectivity of the MOF. Analyzing the Fourier Transform Infrared Spectroscopy (FTIR) spectra of the pristine graphene and graphene-based nanocomposites (GO/UiO-66-NDC), they concluded that the –OH and COO groups of the nanocomposite play an important role in the adsorption of Cr(VI). Moreover, their X-ray photoelectron spectroscopy (XPS) analysis confirms the adsorption of Cr. Using scanning electron microscopy (SEM), they showed that the porous surface of the graphene-based nanocomposite suffers modification as a result of Cr adsorption, which was also confirmed by EDS experiments. Their adsorption studies showed a higher Cr (VI) adsorption capacity of the prepared GO/UiO-66-NDC sample than any other MOFs or nanocomposites reported in the scientific literature (with a value of 157.23 mg g^−1^ of Cr(VI)).

For the water-soluble dye removal from wastewater, we would like to highlight the work done by Liu et al., where they used magnetic graphene aerogels for malachite green dye removal [87]. In order to produce the graphene-based aerogels, they started from Lotus seeds collected from farmers’ markets in Zhengzhou City, Henan Province, China. The first step was to turn the Lotus seeds, following several steps, into lotus seedpod-based activated carbon (LSAC), which was stored in an airtight desiccator. graphene oxide (GO) was dissolved in deionized water and sonicated to obtain a homogeneous aqueous suspension. LSAC was added to the GO solution and stirred for 1 h. CuFe_2_O_4_ was added to the solution and sonicated again. The solution was placed into a 100 mL Teflon-lined autoclave and heated to 90 °C. Magnetic composite graphene aerogels were prepared by freeze-drying the obtained composite hydrogels, yielding the Magnetic Lotus Seedpod-based activated carbon/graphene aerogel composite (MLGA). The MLGA sample was investigated in parallel with the magnetic graphene aerogel (MGA), a sample obtained similarly but without adding LSAC to the preparation. For characterizing the samples, the following techniques were employed: Transmission Electron Microscopy (TEM), SEM, Atomic Force Microscopy (AFM), Brunauer–Emmett–Teller surface area analysis (BET), XPS, XRD, Raman spectroscopy, contact angle measurements, and FTIR. The magnetic properties of the samples were evaluated using a Vibrating Sample Magnetometer (VSM). Adsorption experiments were conducted using the batch method to assess the influence of key operational parameters, including adsorbent dosage, contact time, initial pH, temperature, and initial MG concentration. The variation of adsorption capacity as a function of initial MG concentration demonstrated that MLGA possesses a greater number of available adsorption sites compared to MGA, attributed to the incorporation of LSAC. The initial pH of the MG solution also strongly affects the adsorption capacity, modulating the adsorbent surface charge and the adsorbent–adsorbate electrostatic interactions. An important observation reported by the authors is the very slight decrease in adsorption capacity when changing the pH from 6 to 4, with MLGA still retaining 99.6% of its maximum capacity at pH 4. The optimal conditions for adsorption were established at pH 4, an adsorbent dosage of 0.2 g L^−1^, an initial MG concentration of 100 mg L^−1^, and a contact time of 360 min, yielding a maximum adsorption capacity of 484.3 mg g^−1^. In terms of adsorption mechanisms, the authors proposed several main contributors, including electrostatic attraction, pore filling, hydrogen bonding, and π–π stacking interactions between the aromatic rings of MG and the graphene framework in MLGA.

## 7. Air Purification with Graphene-Containing Materials/Systems

Although the vast majority of the applications concerning the use of graphene and its derivatives in the field of adsorbent materials target the decontamination of water and wastewater sources, there are fruitful results in the field of air purification too [70]. The use of graphene and its derivatives was reported as a good candidate to remove CO_2_, one of the main greenhouse gas producers, from the atmosphere [88].

Graphene-based air purifiers, cutting-edge technology with high impact for air quality, have significantly increased the efficiency in removing pollutants, dust, and allergens with respect to the older versions of air purifiers. These new and improved versions of air purifiers employ a filtration system in multiple steps, one of them being the graphene stage. The graphene layers, due to their excellent properties, trap pollutants more efficiently than other types of filters. Having a nanometric size, graphene is efficient in filtering small particles at the nanometric scale. Its high electrical conductivity helps capture and remove charged particles, while the increased mechanical strength extends the durability of these filters.

CO_2_ is a major contributor to climate change, and several strategies were tested for its removal from flue gases. Adsorption and the use of membranes, cryogenics, and other methods were applied in order to reduce or limit the concentration of CO_2_ in the atmosphere. Adsorbents are easy to regenerate, making them one of the most applied solutions for air purification when talking about CO_2_ as a pollutant. In order to have good efficiency towards the adsorption of CO_2_, adsorbent materials must meet some criteria, such as high adsorption capacity, appropriate selectivity, chemical stability, rapid adsorption/desorption kinetics, etc. Several materials with high potential in CO_2_ adsorption have been reported in the scientific literature: activated carbon, porous inorganic membranes, carbon nanotubes, graphite, carbon spheres, zeolite-based molecular sieves, graphene oxide, and graphene-based adsorbents [89,90].

There are two categories of air filters: porous membranes and fibrous filters [91]. PM (particulate matter) particles are solid or liquid particles suspended in the air. They vary in composition and size depending on the origin (the anthropogenic process producing them) [67,68]. PM_2.5_, composed of particles of up to 2.5 microns in diameter, attacks the cardiovascular system immediately after exposure and poses more health issues than PM_10_, which is composed of larger particles (diameters up to 10 microns). The smaller the particles, the more dangerous they are for human health due to their larger specific surface area, reactivity, mobility, and different compositions. PM_0.3_, for example, which has particles with a size in the order of 300 nm, are very difficult to capture, traveling long distances and carrying with them the danger of transferring bacteria and viruses [92]. Beyond the direct effects on human and animal health, altering it by different diseases, PM particles also have a negative impact on plants, limiting their access to sunlight either by scattering it when floating in the air or by blocking it once they settle on leaves, resulting in poor photosynthesis [93]. Therefore, the need for efficient and effective technological solutions to limit their negative impact is obvious. Porous membranes retain large-sized PM pollutants, while fibrous filters are more efficient for small-sized PM particles.

In order to produce graphene oxide-based membranes for air purification, it is worth following the work of Weiwu Zou et al., described in a paper published in 2019 [71]. They reported graphene oxide preparation using a modified Hummers method, which led to the production of membranes with a large specific surface area and continuous pore structure. They investigated particulate matter removal efficiency as a function of pollutant concentration and wind velocity. The outcome of their work showed high removal efficiency (99.46%) in the case of PM_2.5_ pollutants for a wind velocity of 0.1 ms^−1^. A key point in their study was the preparation of the graphene oxide membrane via a pollution-free method consisting of blade-coating the graphene oxide slurry, obtained by stirring and sonication, onto a nonwoven fabric.

When talking about CO_2_ capturing, we would like to draw attention to two recent scientific publications that deal with CO_2_ capturing. The first one is the work of Navik et al., where they investigated a plasma surface-functionalized graphene adsorbent for CO_2_ capture [72]. They tried to improve the well-known limited adsorption capacity of graphene-based adsorbent materials towards CO_2_ capturing and their selectivity using a N_2_/H_2_ plasma treatment process. The applied plasma treatment increased surface area and textural properties, also enhancing the CO_2_ adsorption capacity from 1.6 to 3.3 mmol/g for flue gas and from 0.14 to 1.3 mmol/g for direct air capture. The adsorption capacity of the plasma-treated aerogels after 40 adsorption/desorption cycles dropped by 17–19%, proving their excellent stability.

A second paper that we highlight, published in 2025, was written by Jerome et al., and describes the results of synthesizing aerogel composites of cellulose with graphene oxide for CO_2_ adsorption [94]. The cellulose-GO aerogel adsorbents functionalized with aminopropyl triethoxysilane (APTES) developed in their work used cellulose extracted from waste paper via alkali and bleaching treatment. At 20 °C and 1 bar, the APTES–grafted hybrid adsorbent was characterized by a CO_2_ adsorption capacity of 2.5 mmol g^−1^, which increased with temperature. The work addresses the dual challenges of upcycling waste paper and CO_2_ emission mitigation.

The removal of hydrogen sulfide (H_2_S) using Cu-BTC, a member of the MOF family, as well as adsorbents with low content (0.5 wt%) of graphene oxide (GO), few-layer graphene (FLG), and thermally reduced graphene (TRG) was investigated by Varghese et al. [95]. Different adsorption capacities were obtained at two temperatures, 298 K and 423 K, and for three different adsorbents. For Cu-BTC/GO, 28.7 and 24.2 mg g^−1^, respectively, were determined for the two mentioned temperatures, while for Cu-BTC/FLG, there was a strong increase up to 44.6 and 53.7 mg g^−1^, respectively. In the case of Cu-BTC/TRG, the values of the adsorption capacities were 44.2 and 26.8 mg g^−1^ at 298 K and 423 K, respectively. All these values are significantly higher than the adsorption capacities of the pure MOF obtained at the mentioned temperatures, i.e., 22.1 mg g^−1^ at 298 K and 21.6 mg g^−1^ at 423 K. In terms of structural changes induced by the different types of graphene-based materials added to the pure MOF, it was observed that graphene increases the pore volume, while GO increases the porosity and the surface area of the composite. Also, adding FLG or TRG to the synthesis has the effect of introducing mesopores to the structure.

## 8. Graphene-Based/Containing Materials for Soil Remediation

Soil, like water and the atmosphere, is too often the silent victim of industrial development due to the rapid increase in the number of hazardous materials used that are, accidentally or not, released into the environment. Population growth, industrial and technological expansion, and the limited use of renewable energy sources have significantly increased environmental pressure. Furthermore, inadequate recycling policies and understanding, along with the slow and costly development of circular economy practices, exacerbate soil contamination. In the case of soil contamination, pesticides, hydrocarbons, heavy metals, and industrial effluents have been identified as the main pollutants [69,73]. The relevance of soil contamination with any toxic compounds is extremely high, affecting the major agricultural component for sustaining life on Earth. Of concern, there are speculations that the increase in the population that is expected to grow beyond 10 billion people by the year 2050 will strongly increase the need for food production, enforcing, in the meantime, unsustainable agricultural practices [96]. Coming with a high adsorption rate into soil provided by the water that plays the role of a binding agent, pesticides strongly affect soil fertility, in general [97]. Petroleum hydrocarbon soil pollution alters crop growth and seed germination, hampering agricultural output [98]. Heavy metals prevent the growth of crop plants, highly limiting their output [99]. For soil pollution, the most noticeable heavy metals are As, Pb, Cd, Hg, Cu, Zn, Cr, Ni, etc. [100].

Similarly to the case of air purification using graphene-based composites/materials, the number of scientific results providing information about the application of this class of materials for soil remediation is considerably fewer than those dedicated to water decontamination processes. One of the recent examples of such results is the contribution of Baragaño et al. from 2020, where graphene oxide nanoparticles, among others, were used for mobilization/immobilization strategies for soil remediation [74]. For the preparation of graphene oxide, the authors used the Hummers–Offerman method, consisting of graphite powder oxidation [101]. The graphene oxide nanoparticles immobilized Cu, Pb, and Cd, while mobilizing As and P from the field soil samples used in the experiments. The pH of the soil was only slightly affected by the use of graphene oxide nanoparticles, decreasing it due to the acidic nature of graphene oxide nanoparticles, in general. The electric conductivity of the samples increased when treated with graphene oxide nanoparticles. This paper also highlighted the option of using combined strategies for soil remediation, recommending, for example, the use of the phytoremediation method in combination with the mentioned graphene nanoparticles.

Following the paper of Mahajan et al., we could divide the graphene-based materials used for soil remediation into several types: graphene, graphene oxide, graphene-based nanocomposites, and functionalized graphene [68]. Pristine graphene, including few-layered graphene materials characterized by unique thermal, mechanical, optical, and electrical properties, was successfully applied in catalytic processes leading to the degradation of different pollutants [76,102].

In the recent publication of Akhtar et al. from 2024, the efficiency in arsenic removal from contaminated soil samples of two differently prepared graphene oxides and magnetite nanoparticles was reported [75]. They prepared graphene oxide via pyrolysis from sugarcane bagasse and via chemical exfoliation, as well as iron oxide nanoparticles using *Azadirachta indica* leaf extract, which were characterized using ultraviolet–visible spectrophotometry (UV–Vis), FTIR, XRD, SEM, and energy-dispersive X-ray spectroscopy (EDX). The sorption tests performed on soils amended with different doses of the three adsorbent materials were prepared and exposed to three different concentrations of As, estimated by calorimetry and atomic absorption spectroscopy. Both graphene-based soil amendment materials exhibit significant arsenic removal/immobilization capacity, from 65 up to 99.3%. This proves that graphene-based soil amendments are promising materials for polluted soil decontamination.

Even more recently, Wang et al. published a paper that optimizes the surface structural properties of 2D graphene oxide with sodium ascorbate and investigated it as a loess soil amendment that could improve the soil in a large region of the Loess Plateau in China [103]. The severe soil erosion specific to this region leads to, among other problems, floods and river siltation. The key problem is the low capacity of the loess soil in general to retain nutrients and water that would help develop more robust structural properties with higher organic content. The addition of graphene oxide reduces the disintegration of the tested loess soils. Moreover, the increase in graphene oxide reduction degree plays an important role in the water retention capacity of the soil, enhancing it for moderate reduction degrees (50%), but at high reduction degrees (100%), the water retention capacity decreases. Nevertheless, this work proves, once again, the possibility of using synergic effects of graphene and various other materials in order to prevent soil erosion and enhance ecological soil restoration. We would expect that this method of soil amendments would be useful, after carefully selecting the combinations of materials involved and fine tuning of the experimental conditions, for other types of soils too.

## 9. Discussion

Although graphene is composed exclusively of carbon, its toxicity depends on several factors, including concentration, particle size, surface chemistry, and exposure conditions. Therefore, special attention should be paid to the possible formation of toxic substances when new composites are prepared through different chemical and physical methods [104,105]. The environmental impact of graphene and its composites should be carefully evaluated, as these materials may alter soil properties through direct and indirect mechanisms. Microbial activity, as well as the availability of nutrients, could be strongly affected by the interaction with naturally occurring components in the soil itself. Nevertheless, the toxicity of any substance greatly depends on two major factors: its properties/composition and its concentration. Even if graphene could be considered non-toxic, at high concentrations, it might have a severe negative impact on both soil and aqueous environments. Those high concentrations are not necessarily obtained in the short term but could also be an obvious consequence of the accumulation process due to its chemical stability.

The question of what happens to the adsorbent after use is one that most laboratory studies leave unanswered. Centrifugation and filtration are standard at the bench scale, but neither scales easily to continuous industrial treatment without significant increases in cost and process complexity. Magnetic composites offer a more practical route, building a magnetic component directly into the architecture, as in the CuFe_2_O_4_-containing MLGA aerogels of Liu et al. [87], which allow for recovery with an external field and retained 99.6% of their adsorption capacity after repeated cycles. The rGO foams of Barra et al. [80] illustrate a different angle: sufficiently low desorption rates mean that the pollutant stays bound after recovery, reducing the risk of secondary contamination in real matrix applications. For gas-phase systems, the recovery problem largely dissolves in fixed-bed configurations, where the adsorbent remains stationary and regeneration is performed in situ. Navik et al. [72] demonstrated this over 40 consecutive cycles, and Zou et al. [71] showed that GO membranes can combine adsorption and physical separation in a single operation. Macroscopic three-dimensional graphene structures offer yet another practical advantage since their bulk form makes mechanical retrieval straightforward in a way that powders and dispersions do not [76]. Despite these examples, no systematic comparison of recovery strategies under realistic operating conditions exists in the literature, and closing that gap is one of the more pressing engineering tasks facing the field.

Looking at the data in Table 3 as a whole, a few things stand out. Pristine graphene and bare GO rarely deliver the best results; what consistently performs better is graphene oxide that has been combined with something else, such as a polymer, a metal oxide, a MOF, or a supporting matrix. The PEI-functionalized composites are the clearest example: 3300.9 mg g^−1^ for malachite green and 313.5 mg g^−1^ for Cr(VI) are numbers that unmodified graphene cannot get close to, and the pattern holds across different pollutant classes. Surface chemistry, more than surface area, seems to be doing most of the work. The three compartments are not equally well covered. Water has, by far, the most entries, the widest variety of materials, and the most developed experimental methodology. Soil is at the other end. There are useful results, but the studies tend to focus on immobilization rather than removal in the classical sense, and field conditions introduce enough variability that comparing performance across papers is difficult. Air sits somewhere in between, with genuinely promising CO_2_ capture and PM removal results, but with a narrower range of materials tested. The limitation that cuts across all three is the same one that comes up repeatedly in the individual study descriptions: single-pollutant systems, controlled pH, and clean water matrices. These conditions make it easy to measure adsorption capacity, but they do not tell you much about what would actually happens in a treatment plant.

Adsorption is only one of the roles these materials play in environmental remediation. The same graphene derivatives are typically multifunctional [23]. The clearest overlap is with photocatalysis, where composites such as rGO/ZnO or rGO–N-CaTiO_3_ work as an adsorbent and photocatalyst at the same time, so the pollutant gathered at the surface is also broken down rather than merely held [106,107]. Membrane-based treatment is a second direction. Graphene oxide layers can combine adsorption with physical separation in a single step, as already shown for air purification [71]. What ties these together is that they draw on the same surface properties that this review emphasizes for adsorption, which is why advances in graphene adsorbents usually carry over to the wider set of environmental applications.

Further investigations regarding the applications of graphene in general will certainly cover the area of computer simulations. One of these kinds of examples is the work of Slepchenkov et al., where the authors used density functional theory-based calculations in order to evaluate the possibility of using island-type graphene-nanotube films in stretchable electronic devices [108]. Their predictive atomic models evaluate the changes in the electrically conductive properties of the discussed samples. The investigated hybrid films consisted of AB-stacked graphene and chiral single-walled carbon nanotubes with chirality indices (12.6) and a diameter of 1.2 nm. The graphene bilayer was placed on top of the carbon nanotube, increasing the carbon density, as in the case of experimentally synthesized graphene-nanotube composites. The authors found that the resistance to failure of the graphene bilayer-nanotube composite is higher in the case of axial compression with respect to axial stretching. After optimization of the supercells formed by a graphene bilayer, carbon nanotube composite, both the bilayer and the nanotube underwent deformation, although the topological models considered were proven to be energetically stable [109].

We are confident that given the strong increase in the processing power of computers, combined with the promising results brought by the evolution of artificial intelligence, similar studies will appear in the extremely complex field of graphene-based adsorbents applied to air, soil, and water pollutants. An example is the paper by Rangel-Vazquez et al., which contains details about a dispersion-corrected DFT investigation of the impact of oxygen-containing functional groups on the optical and electronic properties of graphene. The authors concluded that the graphene functionalization using oxygenated functional groups (COO, CO, or OH) increased the active sites that could anchor Li ions from the solution. Another theoretical investigation is the work of Tabari and Farmanzadeh from 2019, where they used DFT calculations to investigate Y-doped graphene oxide for the adsorption of ethylene, water, and carbon monoxide [110]. They arrived at the conclusion that doping with Y leads to an increase in the adsorption energies for the studied adsorbed materials.

Beyond DFT, a faster-moving development over the last few years has been the use of machine learning (ML) and artificial intelligence to predict adsorption performance directly from material and process descriptors, which is exactly the kind of screening that could accelerate the search for better graphene-based adsorbents. Al-Jamimi et al. built hybrid AI models that predicted the removal of dyes and phenols by an amine-functionalized graphene oxide/layered triple hydroxide composite with high accuracy [111]. Regarding gas, Fathalian et al. trained seven algorithms on data extracted from the literature and reproduced the CO_2_ uptake of GO-based sorbents, with a multilayer-perceptron network reaching R^2^ above 0.99 [112]. More recent work has turned to the practical obstacles: Tran et al. compared several methods on the small datasets that are typical of graphene adsorption studies and found that support vector machines and TensorFlow-based networks tolerate limited data better than polynomial regression [113], while Zhai et al. used interpretable and generative models, including data augmentation, to predict the simultaneous removal of organic pollutants and heavy metals when experimental data is scarce [114]. The recurring limitation across these studies is the data itself: adsorption datasets are small and inconsistently reported, so the immediate priority is less a matter of more elaborate algorithms than of larger, standardized datasets on which they can be trained.

What ultimately decides whether any of these materials get used outside the lab is cost, and the two routes people discuss most are far apart on that front. CVD is the most costly one. Growing graphene at around 1000 °C on a metal catalyst under vacuum, then transferring the film off that catalyst, gives excellent material but very little of it, and what you end up with is a film bound to a substrate rather than the loose powder an adsorbent is built from. Flash Joule heating goes the other way. Luong et al. showed that a single electric pulse lasting under a second can turn cheap carbon, or even waste such as coal, petroleum coke, food waste, and mixed plastic, into gram amounts of turbostratic graphene. They put the electricity cost at about 7.2 kJ per gram, or roughly USD 100 per ton of graphene, at a time when graphene itself sold for somewhere between USD 67,000 and USD 200,000 per ton [115]. At the scale of a real remediation project, that gap matters. FJH gives the dispersible powder that composite adsorbents are made from; its feedstock is often a waste stream that would otherwise need disposing of, and it avoids the furnaces, solvents, and transfer steps that make CVD so hard to run at ton scale. Flash graphene is turbostratic and not as structurally clean as CVD film, but adsorption depends on surface area and accessible functional groups far more than on crystalline perfection, so here, that is not much of a loss.

Another issue that is often overlooked is the environmental footprint associated with graphene production itself. High adsorption performance does not automatically translate into a sustainable remediation technology. This is particularly relevant for graphene oxide, which is commonly produced through modified Hummers-type methods involving strong oxidizing agents and concentrated acids. Recent Life Cycle Assessment (LCA) studies have shown that the environmental benefits of graphene-based systems depend not only on pollutant removal efficiency, but also on synthesis conditions, energy consumption, waste generation, and material recovery strategies [116,117]. Consequently, the practical value of graphene-based remediation technologies should be assessed across their entire life cycle rather than solely through adsorption performance metrics.

**Table 3:** Summary of representative graphene-based adsorbents reported for environmental applications, including the adsorbent used, pollutant removed, adsorption performance, and reference.

Adsorbent	Pollutant	Performance	Reference
rGO@ZnO	Rhodamine B(RhB)	Improved RhB adsorption versus neat ZnO; 99% recovery over several reuse cycles under simulated sunlight	[106]
rGO–N-CaTiO_3_ composite	Methylene blue	Enhanced adsorptive-photocatalytic removal versus bare materials; stable and recyclable	[107]
ZnFe_2_O_4_@rGO magnetic nanocomposite	Pb(II)	89.8 mg g^−1^ adsorption capacity	[118]
ZnFe_2_O_4_@rGO magnetic nanocomposite	Methylene blue	119.0 mg g^−1^ adsorption capacity; reusable for five cycles	[118]
PG@GO@PEI	Cr(VI)	Removal rates close to 100%; maximum capacity 313.5 mg g^−1^	[119]
PG@GO@PEI	Naphthol Green B	Removal rates close to 100%; maximum capacity 425.5 mg g^−1^	[119]
PG@GO@PEI	Malachite Green	Removal rates close to 100%; maximum capacity 3300.9 mg g^−1^	[119]
Graphene	Perchlorate	99.2% adsorption efficiency; capacity up to 0.024 mg g^−1^	[120]
Graphene	Methylene blue	Maximum adsorption capacity of about 1.52 g g^−1^; stable for five cycles	[120]
Graphene	p-toluenesulfonic acid	Maximum adsorption capacity of about 1.43 g g^−1^	[120]
Graphene	1 1-naphthalenesulfonic acid	Maximum adsorption capacity of about 1.46 g g^−1^	[120]
GO-ordered mesoporous silica composite	As	97.7% removal efficiency	[121]
GO-ordered mesoporous silica composite	Cd	96.9% removal efficiency	[121]
GO-ordered mesoporous silica composite	Cr	96.0% removal efficiency	[121]
GO-ordered mesoporous silica composite	Hg	98.5% removal efficiency	[121]
GO-ordered mesoporous silica composite	Pb	78.7% removal efficiency	[121]
GO	Ciprofloxacin	Adsorption capacity of 500 mg g^−1^	[77]
GO	Zn(II)	Adsorption capacity of 345 mg g^−1^	[77]
GO	Methylene blue	Adsorption capacity of 450 mg g^−1^	[77]
GO	Neonicotinoids	Adsorption capacity of 3.11 mg g^−1^	[77]
Graphene-based aerogels	Pb, Zn, Cd	Reported adsorption efficiencies of 367, 246, and 106.3 mg g^−1^, respectively	[122]

PG@GO@PEI—phosphogypsum (PG) with graphene oxide (GO) and polyethyleneimine (PEI); rGO—reduced graphene oxide.

Table 3 shows that the most effective systems are generally PEI-functionalized graphene oxide composites, particularly when combined with a supporting matrix such as phosphogypsum or polyvinyl alcohol. Among the compared materials, PG@GO@PEI showed outstanding dye-removal performance, reaching 3300.9 mg g^−1^ for malachite green. These findings suggest that PEI-modified graphene-based composites are among the most promising candidates for environmental adsorption applications, especially in wastewater treatment. For comparison, conventional activated carbon, still the benchmark commercial adsorbent, relies mainly on surface area and porosity [90], and for these dyes and metals, it generally reaches lower capacities than the tailored graphene composites in Table 3. This reinforces the point that surface chemistry, more than surface area, is what sets the leading materials apart.

Something that tends to get lost in the focus on adsorption numbers is what happens to these materials afterwards. Graphene composites are not inert. Once they leave the laboratory, they end up in soil, water, and organisms, and their chemical stability, which is an asset during adsorption, becomes a liability when thinking about long-term accumulation. The toxicological picture is still genuinely unclear for most of these composites. Graphene oxide behaves differently from rGO, which behaves differently again from a PEI-functionalized composite, and very few studies have tracked what any of them do in a real ecosystem over time. This is not a reason to stop developing these materials, but it is a reason to take the safety data more seriously than the field currently does, and to treat ecotoxicological characterization as a standard part of the workflow rather than an afterthought.

## 10. Conclusions

More than two decades have passed since graphene was first experimentally isolated, yet its trajectory in the scientific community remains difficult to assess in simple terms. Comparing its evolution with that of other landmark materials, it becomes evident that early expectations were frequently shaped by an enthusiasm that outpaced the realities of industrial development and large-scale implementation.

This pattern is not unique to graphene. The history of materials science is marked by periods of intense excitement around discoveries, followed by a more measured reassessment of their actual potential. What sets graphene apart, however, is that even after this reassessment, it continues to demonstrate genuine relevance across a remarkably broad range of fields. New applications and derivative materials are still being reported at a sustained pace, and the scientific community shows no signs of exhausting the possibilities offered by this material and its composites.

Some of the most obvious gaps have to do with scaling. Obtaining good adsorption numbers in a lab flask is one thing, but building a system that works continuously, handles real contaminated water or air, and can be maintained without specialist input is something else entirely. Most of the synthesis methods that give the best material quality are also the hardest to scale, CVD being the obvious example, and that tension has not been resolved in two decades of research. The environmental fate of these materials is another area where the literature is thinner than it should be. Graphene composites are chemically stable by design, which is useful for adsorption but means they persist in soil and water long after use, and very few studies have followed what happens to them under realistic environmental conditions over time. On the more practical side, most of the reviewed adsorbents were tested on one pollutant at a time under controlled conditions, which tells you relatively little about how they would behave in a real effluent with competing ions, variable pH, and organic matter. Computational tools, DFT in particular, are starting to make it easier to predict which modifications will actually help before committing to synthesis, and that is probably where some of the most useful near-term progress will come from.

None of this means that the field is going in the wrong direction. The range of materials, applications, and performance levels documented in this review is impressive, and there is no shortage of interesting directions to pursue. However, the next meaningful step is probably not another high-capacity adsorbent demonstrated in a clean single-pollutant system, but is closer to figuring out which of the existing materials actually hold up under real conditions, can be recovered and reused without falling apart, and can be produced at a cost that makes sense outside of a research context. This is a harder problem than optimizing surface chemistry, but it is the one that matters if any of this work is going to move beyond the journal page.

## Figures and Tables

**Table 1:** Summary of the benefits and weaknesses of the previously described graphene production methods.

Graphene Preparation Method	Advantages	Drawbacks
Scotch tape (micromechanical exfoliation)	- High-quality material (graphene) - Low cost and very simple - Does not require specialized equipment	- Not scalable at the industrial level - Low production yield (<1%)
Chemical Vapor Deposition (CVD)	- High-quality material (graphene) - Large area samples produced - Much higher yield than the scotch tape method (10–30%)	- Requires relatively expensive specialized equipment - Complex process (specialized workforce required)
Thermal Decomposition of SiC	- High-quality graphene - Low number of defects - Relatively controllable thickness	- Expensive due to SiC substrates and high temperatures required - Difficult to control the number of layers over a large surface area
Liquid Phase Exfoliation (LPE)	- Simple to apply - Relatively easily scalable for large quantities of production	- Can introduce defects in the final product - Easy to get the final product contaminated by surfactants - There are variations in the thickness of the resulting graphene
Oxidation– reduction technique	- The high dispersibility of graphene oxide in solvents helps perform this method - Easily scalable to large quantities	- Concerns related to the environmental impact of the chemicals used - Lower electrical conductivity of the GO/rGO final product compared to other types of (pristine) graphene
Flash Joule Heating (FJH)	- Low costs and simple to apply - Applicable for a large variety of carbon sources on a large scale - Relatively high production yield (30–60%)	- Lower quality than the CVD-produced graphene

**Table 2:** The most relevant properties of graphene as a function of its layers.

Properties	SLG	DLG	MLG	GO
Electron mobility [cm^2^ V^−1^ s^−1^] [49,50,51,52]	2 × 10^5^	10^5^	300–1150	200
Specific surface area [m^2^ g^−1^] [53,54,55,56]	2630	1300	270–1500	750–2400
Thermal conductivity [W m^−1^ K^−1^] [57,58]	5000	1900	1100	70–670
Young’s modulus [GPa] [59,60,61]	2400	2000	1850	32

SLG—Single-layer graphene; DLG—Double-layer graphene; MLG—Multiple-layer graphene; GO—Graphene oxide.

## Data Availability

No new data were created or analyzed in this study. Data sharing does not apply to this article.
